# Nitrogen Dissolution in Liquid Ga and Fe: Comprehensive *Ab Initio* Analysis, Relevance for Crystallization of GaN

**DOI:** 10.3390/ma14051306

**Published:** 2021-03-09

**Authors:** Jacek Piechota, Stanislaw Krukowski, Petro Sadovyi, Bohdan Sadovyi, Sylwester Porowski, Izabella Grzegory

**Affiliations:** Institute of High Pressure Physics, Polish Academy of Sciences, 29/37, Sokolowska Street, 01-142 Warsaw, Poland; jpa@unipress.waw.pl (J.P.); stach@unipress.waw.pl (S.K.); pedro@unipress.waw.pl (P.S.); bsad@unipress.waw.pl (B.S.); sylvek@unipress.waw.pl (S.P.)

**Keywords:** *ab initio*, calculations, nitrogen solubility, liquid gallium, liquid iron, gallium nitride

## Abstract

The dissolution of molecular nitrogen in Ga and Fe was investigated by *ab initio* calculations and some complementary experiments. It was found that the N bonding inside these solvents is fundamentally different. For Ga, it is between *Ga4s* and *Ga4p* and *N2p* states whereas for Fe this is by *N2p* to *Fe4s*, *Fe4p* and *Fe3d* states. Accordingly, the energy of dissolution of N${}_{2}$ for arbitrarily chosen starting atomic configurations was 0.535 eV/mol and −0.299 eV/mol for Ga and Fe, respectively. For configurations optimized with molecular dynamics, the difference between the corresponding energy values, 1.107 eV/mol and 0.003 eV/mol, was similarly large. Full thermodynamic analysis of chemical potential was made employing entropy-derived terms in a Debye picture. The entropy-dependent terms were obtained via a normal conditions path to avoid singularity of ideal gas entropy at zero K. Nitrogen solubility as a function of temperature and N${}_{2}$ pressure was evaluated, being much higher for Fe than for Ga. For T=1800 K and $p=10^{4}$ bar, the N concentration in Ga was $3\times 10^{-3}$ at. fr. whereas for Fe, it was $9\times 10^{-2}$ at. fr. in very good agreement with experimental data. It indicates that liquid Fe could be a prospective solvent for GaN crystallization from metallic solutions.

## 1. Introduction

Growth of large-size single crystals of GaN has been an important issue for the development of semiconductor technology for several decades. That is because GaN is difficult to grow, both from vapor and liquid phases, and to dope effectively to *n*-type, *p*-type and semi-insulating. A number of obstacles contribute to these difficulties, including thermodynamic and kinetic factors [1].

GaN is required for the manufacture of a wide variety of optoelectronic and electronic semiconductor devices, such as optoelectronic light-emitting diodes (LEDs) and laser diodes (LDs) [2], to recently developed THz emitters [2], or in electronic high-power transistors, high electron mobility transistors (HEMT) [3], or 2-D gas (2DEG)-based molecular sensors [4]. The performance of these devices critically depends on the quality and the properties of substrates used for epitaxy. Thus, the goal is to develop reliable and cost-efficient methods of crystallization of large-size excellent-quality single crystals. Despite considerable progress attained using several methods, including gas-phase hydride vapor-phase epitaxy (HVPE) [5,6], ammonothermal [7], or solution growth [8], the goal was only partially achieved.

Generally, liquid-phase methods are more suitable for providing high-quality crystals than those based on the vapor source, as the temperature gradients are easier to control in the liquid, the control of point defects is better, and the chemical purity is easier to protect [9,10]. The gas-phase growth of GaN does not include direct synthesis from the elements, as the kinetics blocks the process thus additional transport agents contribute to uncontrolled doping. The liquid-phase growth includes ammonia-based and metal-based methods. The ammonia-based method entails the application of supercritical ammonia as a solvent which necessitates the use of group I element as a transport vehicle for gallium [7]. This leads to inevitable uncontrolled doping of the grown crystals, which is very harmful. At the same time, the crystals are of exceptionally good crystallographic quality, confirming the expectance related to the liquid-phase methods. The metal-based methods include the high nitrogen pressure solution growth (HNPSG) using pressures of order of 10 kbar to attain nitrogen solubility of 1 at.%, necessary for effective crystallization of GaN from liquid gallium [11]. The high gas pressure limits the possible size of the growth apparatus which causes numerous technical problems, and leads to the limited size of the resulting GaN crystals. On the other hand, these crystals were of exceptionally good quality, again in agreement with the liquid-phase methods expectancy [11]. However due to technical limitations, at present, the HNPSG method is not suitable for wide industrial applications.

The route for effective dissolution of nitrogen is to overcome the kinetic barrier of N${}_{2}$ dissociation by using metals that catalyze decomposition of nitrogen molecules. Such catalytic influence was identified by Romanowski et al. for dissolution of molecular nitrogen in liquid Ga, Al and In [12,13]. Thus, the successful GaN growth method should assure that nitrogen solution within the liquid metals must be atomic. On the other hand, for metallic solvents other than gallium, the possible metal nitride should be significantly less stable than GaN so that the solvent metal nitride should not compete with GaN during growth. Several such candidates were proposed, including sodium or potassium [8]. These metals were shown to catalyze N${}_{2}$ molecules decomposition and accordingly they were used as solvents in relatively successful GaN growth procedures.

Other possible solvents include transition metals, such as Co, Ni or Fe [14,15]. These metals were and still are successfully used in high-pressure solution growth of diamonds [16,17]. The use of Fe is the very basis of extremely successful Sumitomo method of growth of high-quality diamonds [18]. It has been shown experimentally [14,15] that significant concentrations of nitrogen in the transition metals can be achieved at pressure and temperature conditions relevant for the solution growth of GaN. Thus, iron has high propensity to nitrogen and could potentially catalyze the nitrogen molecule decomposition and its incorporation into GaN crystalline lattice. In addition the solvent should include some percentage of gallium, necessary to build the GaN crystal.

The present paper is devoted to extensive theoretical investigations of basic properties of Ga-N and Fe-N systems potentially interesting for the growth of GaN single crystals under high nitrogen pressure using Fe-Ga solvents. A verification of the theoretical predictions by both existing and new experimental data obtained within this study, is also presented and discussed. The investigation includes atomic level modeling of the thermal contributions to gas-liquid equilibria expressed as equality of chemical potential of nitrogen in these phases. The essential part of the study are *ab initio* calculations for the Fe-N and Ga-N systems, allowing determination of the course of nitrogen dissolution processes in the liquid metals and the thermodynamic properties of the corresponding phase systems. The full pressure–temperature equilibrium diagrams for the two relevant systems are evaluated.

It is worth underlining that the essential goal of the paper is to explore the applicability of the *ab initio* methods in determination of the pressure–temperature equilibrium diagrams. As it will be shown, the essential part of the study is the determination of the chemical potentials of the dissolved species. Then the pressure is obtained by using exponential functions of the argument that is temperature-dependent, and additionally, divided by the temperature. Thus, the nature of the functional dependence indicates that the errors in the pressure dependence may be enhanced in highly nonlinear way. Therefore, the pressure–temperature dependencies of the chemical potentials may be recovered with some error only, due to the different error sources in both the DFT method itself and its implementation in the thermodynamic calculations as discussed below.

## 2. Methods

### 2.1. Calculation Method

Density functional theory (DFT)-based code SIESTA [19,20] was employed in all *ab initio* calculations reported in this study. SIESTA is the code developed under Spanish Initiative for Electronic Simulations with Thousands of Atoms which was designed to simulate systems including thousands of atoms. In the present calculations the more precise version, capable of dealing with smaller number of atoms, was used. The solution procedure used in Generalized Gradient Approximation (GGA) employs norm-conserving pseudopotentials for determination of the system wave-function created using linear combinations of local basis molecular orbitals. All electron calculations are not possible, therefore the norm-conserving Troullier–Martins pseudopotentials, in the Kleinmann–Bylander factorized form [21,22] for Ga, Fe and N atoms were generated using ATOM program written by the authors of the SIESTA code. The exchange-correlation functional was adopted in revised modification of Perdew, Burke and Ernzerhof (PBE) [23,24] functional for solids and surfaces (PBEsol) [25,26].

The cutoff value in the equivalent plane wave for the real space properties and integration grid maximum values was 275 Ry, roughly equal to the grid spacing in the representation of the distribution of any quantity in position space of about 0.1 Å. The solution of Kohn-Sham equations uses finite size atomic orbitals, therefore it is not affected by the grid size. This grid selection affects the real space representation of the properties derived from the grid. The self-consistent field (SCF) loop was terminated when the maximum difference between the output and the input of each element of the density matrix was below 10${}^{-4}$. In the relaxation procedure, the positions of atoms were modified when the forces acting on these atoms were higher than 0.005 eV/Å.

The data obtained in this paper also include an assessment of the number of configurations that are used in the entropy determination. As it is shown, not every configuration is considered, thus the results are also burdened by some systematic error. Thus, the entire chemical potential dependence includes the systematic errors that are enhanced in the evaluation of the equilibrium pressure.

The simulations of interaction between Ga, Fe and N needed critical tests. One of the possible tests was a comparison of DFT results and the experimental data for gallium nitride. The lattice constants following from the *ab initio* total energy minimization of the wurtzite crystalline lattice of GaN were a=3.194 Å, c=5.186 Å. These values are in a reasonable agreement with the experimental data for GaN a=3.1890±0.0003 Å, c=5.1864±0.0002 Å [27].

**Figure 1:**
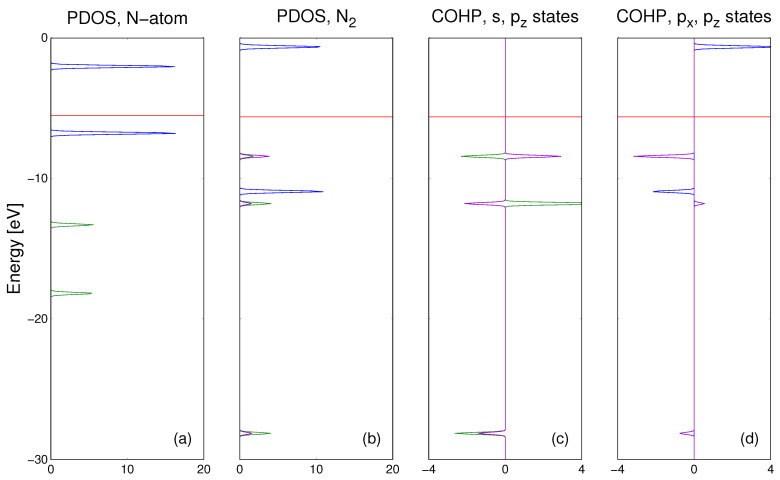
Properties of nitrogen quantum states obtained in this study, by *ab initio* method: (**a**) projected density of states (PDOS) of the separate nitrogen atoms (N), green and blue line represent *N2s* and *N2p* states, respectively; (**b**) PDOS of the nitrogen molecule (N${}_{2}$), green, blue and magenta line represent *N2s*, *N2p${}_{x}$ & N2p${}_{y}$*, and *N2p${}_{z}$* states, respectively (**c**) Crystal Orbital Hamiltonian Population (COHP) of the *N2s-N2s* (green line) and *N2s-N2p${}_{z}$* (magenta line) states, (**d**) COHP of *N2p${}_{x}$-N2p${}_{x}$* (blue line) and *N2p${}_{z}$-N2p${}_{z}$* (magenta line) states. The results are obtained for spin-polarized calculations, so the two peaks correspond to different spin orientations. Horizontal red lines denote Fermi level: $E_{F}=-5.634$ eV.

In addition, the DFT *ab initio* simulations of the nitrogen molecule (N${}_{2}$) characteristics could serve as the test of compatibility of the DFT parameterization for molecular species. This is important as the typical choice for simulation of solids and semiconductors in particular, works poorly for small, covalently bonded molecules. The compromise solution is naturally inferior from the ones optimized for molecules only, and naturally less precise than advanced *ab initio* calculations, such as W1 or CCSD(T) [28,29,30]. The test values for the N${}_{2}$ molecule, obtained in our approach were dissociation energy $\Delta E_{diss}$ and the bond length *d*. The N${}_{2}$ dissociation energy obtained in the presently used parameterization is $\Delta E_{DFT}^{diss}\left(N_{2}\right)=9.801$ eV which is in a good agreement with the experimental value $\Delta E_{exp}^{diss}\left(N_{2}\right)=9.790$ eV [30]. The DFT bond length $d_{DFT}^{N-N}=1.092$ Å was in an excellent agreement with the experimental value $d_{exp}^{N-N}=1.097$ Å [28]. An additional insight may be obtained from the diagram of bonding states in N${}_{2}$ molecule, presented in Figure 1.

The Crystal Orbital Hamiltonian Population (COHP) data is used to determine the bonding/antibonding interactions [31]. The data presented in Figure 1 indicate that the nitrogen *N2s* bonding and antibonding states have the lowest energy and accordingly, they are occupied. In addition the bonding *N2p* states (both 2π and 2σ bonds) are occupied as well so that they contribute to high dissociation energy of the molecule. The integration of COHP peaks gives the following data: (i) $E_{N2s-N2s}=4.002$ eV; (ii) $E_{N2s-N2p_{z}}=2.366$ eV; (iii) $E_{N2p_{z}-N2p_{z}}=1.623$ eV (iv) $E_{N2p_{xy}-N2p_{xy}}=1.616$ eV. These values of the sum of the covalent interaction overlaps is 9.607 eV which is in good agreement with the N${}_{2}$ dissociation energy $\Delta E_{exp}^{diss}\left(N_{2}\right)=9.790$ eV [30].

As a next testing step, basic physical properties of pure gallium and iron were determined. These properties include the cohesive energies, the band structure and the work functions. The atomization energy of gallium, obtained from *ab initio* calculations was $\Delta E_{DFT}^{atom}$(Ga) = 2.898 eV/atom which is in a reasonable agreement with the atomization energy of rhombic Ga structure, equal to $\Delta E_{exp}^{atom}$(Ga) = 2.81 eV/atom as determined in reference [32]. The density of Ga was evaluated as equal to $\rho_{DFT}$(Ga) = 6.304 g/cm${}^{3}$, which is favorably compared to $\rho_{exp}$(Ga) = 6.095 g/cm${}^{3}$ at the melting point (close to 300 K). In summary, the properties of metallic Ga are reasonably recovered by the parameterization of Ga atom orbitals used in SIESTA.

Similar analysis could be made for iron bonding properties. The atomization energy of Fe, obtained from spin-polarized *ab initio* calculations, was $\Delta E_{DFT}^{atom}$(Fe) = 4.978 eV/atom which is in reasonable agreement with the experimental value of $\Delta E_{exp}^{atom}$(Fe) = 4.28 eV/atom [32]. The DFT density of Fe was equal to $\rho_{DFT}$(Fe) = 7.825 g/cm${}^{3}$, which is not far from $\rho_{exp}$(Fe) = 7.874 g/cm${}^{3}$ of iron at normal conditions.

**Figure 2:**
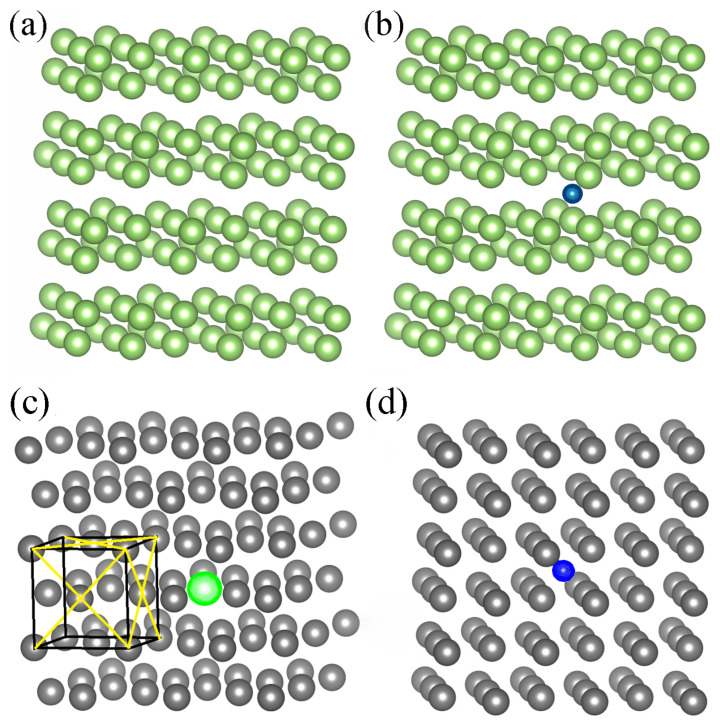
Supercells used for determination of properties of metals: (**a**) 144 Ga atom supercell representing orthorhombic lattice, (**b**) Configuration of 144 Ga atom supercell with immersed single interstitial N atom. The green and blue balls denote Ga and N atoms, respectively. (**c**) Configuration of 108 Fe/Ga atom cluster: 107 Fe atoms and incorporated single Ga atom. The Ga atom replaced one of the Fe atoms in the lattice. The gray and green balls denote Fe and Ga atoms, respectively. (**d**) The configuration of 108 Fe atoms cluster: Fe cluster with immersed interstitial single N atom. The gray and blue balls denote Fe and N atoms, respectively.

For evaluation of Ga and Fe metal properties, the supercells have been chosen. They are shown in Figure 2. Gallium was simulated using 144 Ga atoms supercell (Figure 2a) representing orthorhombic solid Ga lattice, the most stable Ga structure at low temperatures. Iron was simulated using 108 atom supercell representing face centered cubic crystalline lattice. A cluster of 108 atoms of pure Fe is similar to the cluster shown in Figure 2c with the difference that the Ga atom is substituted by the Fe atom (substitution of the green ball to the gray ball).

### 2.2. Experimental

For experimental evaluation of N solubility in Fe at high nitrogen pressure (HP) and at high temperature (HT), a series of annealing experiments in HP-HT gas (N${}_{2}$) pressure reactors was performed. In this study, the pressure dependence of N solubility in the Fe metal liquid, with the N${}_{2}$ gas as a source of the solute, has been determined.

**Figure 3:**
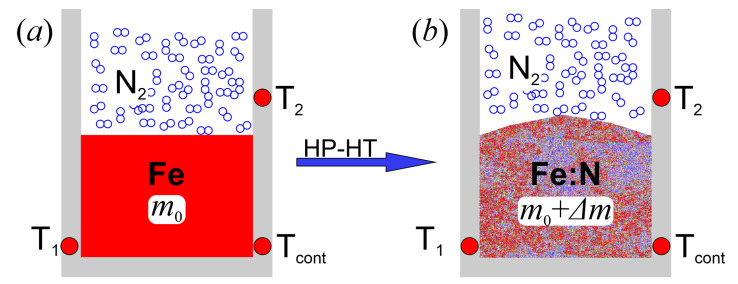
Scheme of sample configuration (cross-section) before (**a**) and after (**b**) the HP-HT annealing run.

For this purpose, a series of iron samples (ARMCO Pure Iron, Grade 2) in the form of cylinder (11 mm in diameter) inserted into BN crucible as shown schematically in Figure 3a was used. Initial mass for each sample (m${}_{0}$) was measured. Then the crucible with the sample was placed into the two-zone furnace and loaded into the high-pressure chamber. The system was evacuated, filled with nitrogen (6 N) and the gas compressed until a required pressure. This preparatory procedure was used in all experiments. The annealing experiments were performed at quasi-isothermal conditions. Annealing temperature for all experiments was 1708 ± 1 K, duration of the annealing at constant temperature was 0.5 h. The system was heated with a rate 800 K/h and cooled by quenching (rate of cooling ca. 3000 K/h) to freeze the high temperature state of the Fe-N solution. The N${}_{2}$ pressure sequence of 5000, 6000, 7000, 8000, 9800 bar was used to determine the pressure dependence of N solubility in the Fe metal.

After the experiments, all samples changed their shape and mass (see scheme in Figure 3b). It was assumed that the increase of the sample mass was due to N dissolved in the metal. A background for such an assumption was our previous analysis reported in references [15,33]. For the reported analysis, the composition of the Fe-N samples treated in a similar way as in this work was studied by the Energy Dispersive X-ray Spectroscopy (EDX—ZEISS SEM with BRUKER detector, Oberkochen, Germany) and the Inert Gas Fusion spectroscopy (ON836 LECO, St. Joseph, MI, USA). We have shown that the observed increase of the sample mass corresponded exactly to the mass of dissolved nitrogen determined by the indicated spectroscopy methods.

The results obtained in this work were used in Section 3.5 for comparison to theoretically calculated solubility values.

## 3. Results and Discussion

### 3.1. Basic Properties of Pure Ga, Fe and Mixed Metals from Ab Initio Calculations

**Figure 4:**
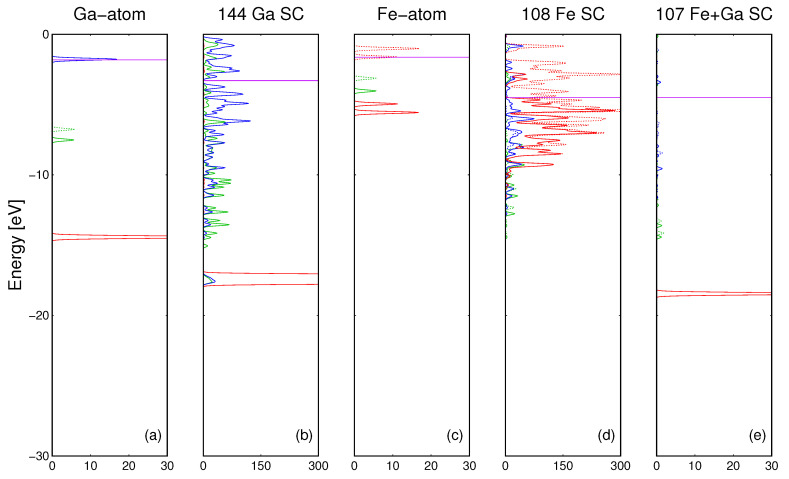
*Ab initio* obtained PDOS of quantum states: (**a**) of single gallium atom (Ga), (**b**) of the supercell (SC) consisting of 144 Ga atoms (green, blue and red line represent *Ga4s*, *Ga4p* and *Ga3d* states, respectively), (**c**) of single iron atom (Fe), (**d**) of the supercell (SC) consisting of 108 Fe atoms (green, blue and red line represent *Fe4s*, *Fe4p* and *Fe3d* states, respectively (**e**) of supercell consisting of 107 Fe and single Ga atom (green, blue and red line represent *Ga4s*, *Ga4p* and *Ga3d* states, respectively) The results are obtained for spin-polarized calculations, so the two peaks correspond to different spin orientations, which are marked by solid and dashed lines respectively. Fermi level is denoted by horizontal magenta line.

The properties of electronic bonding in the pure Ga metal may be compared by plots of DOS of single Ga atom and the Ga supercell consisting of 144 atoms (Figure 2a). The results of the corresponding *ab initio* calculations are presented in Figure 4a,b. As it is shown, the bonding in gallium arises from the extensive overlap of the *Ga4s* and *Ga4p* orbitals. The *d* states of Ga atoms do not participate in the metallic bonding in Ga.

For iron, the electronic bonding may be analyzed by plots of DOS of a single Fe atom and the Fe supercell consisting of 108 atoms. The results are compared in Figure 4c,d. As it is shown, bonding between the iron atoms arises mainly from the extensive overlap of the *Fe3d* orbitals. This is drastically different from Ga where *3d* orbitals do not participate in the bonding. In summary, the properties of metallic Fe show important differences with respect to the Ga bonding.

Finally, a single Ga atom immersed in the Fe matrix was modeled using total 108 atom cluster, composed of 107 Fe atoms and of a single Ga atom. The cluster with Ga atom incorporated is presented in Figure 2c whereas the cluster of pure Fe is inferred from Figure 2c by replacement of Ga by Fe atom (substitution of green by gray ball).

The results of the calculations are shown in Figure 4e. As it is shown, the *3d* states of Ga atoms do not participate in the bonding with Fe matrix. Also *Fe3d* states are not involved. The bonding is due to interaction of Fe and Ga *4s* and *4p* states. The difference is due to different behavior of *Ga3d* states which remain unaffected by surrounding Ga and Fe neighbors. This is demonstrated by the PDOS of Ga/107 Fe SC presented in Figure 4e where *Ga3d* preserved its molecular character (sharp line), not affected by the overlap with the neighboring Fe atoms.

The energetic effect of dissolution of single Ga atom in Fe was calculated taking into account that an Fe atom is replaced by a Ga atom, according to the formula:

(1) $$\Delta E_{DFT}^{dis}\left(\mathrm{Fe}-\mathrm{Ga}\right)=E_{DFT}\left(107\;\mathrm{Fe}-1\;\mathrm{Ga}\right)-E_{DFT}\left(108\;\mathrm{Fe}\right)+E_{DFT}\left(\mathrm{Fe}\right)-E_{DFT}\left(1\;\mathrm{Ga}\right)$$

where $E_{DFT}$(107 Fe-1 Ga) denotes total DFT energy of the cluster of atoms (numbers and symbols denote the cluster size and the type of constituting atoms). From the performed DFT calculations, the energy of Ga dissolution in the Fe matrix was determined as $\Delta E_{DFT}^{dis}$(Fe-Ga) = 3.698 eV/atom. The value is relatively large indicating strong interaction between Ga and Fe atoms. The experimental data on liquid Fe:Ga solutions confirm qualitatively, these results by a drastic decrease of Fe melting temperature induced by small concentrations of gallium in iron [15].

### 3.2. Interaction of N Atom Immersed in the Metal (Ga, Fe) Periodic Clusters

Similar analysis could be made for dissolution of nitrogen in both Fe and Ga solvents. As shown recently by Ponomareva et al., the calculated N dissolution energies in a solid Fe cluster depend on the cluster configuration [34]. The pure Fe cluster could be inferred from Figure 2c by replacement of Ga by Fe atom while Fe cluster with a single immersed N atom is presented in Figure 2d. These clusters are used in our *ab initio* calculations.

**Figure 5:**
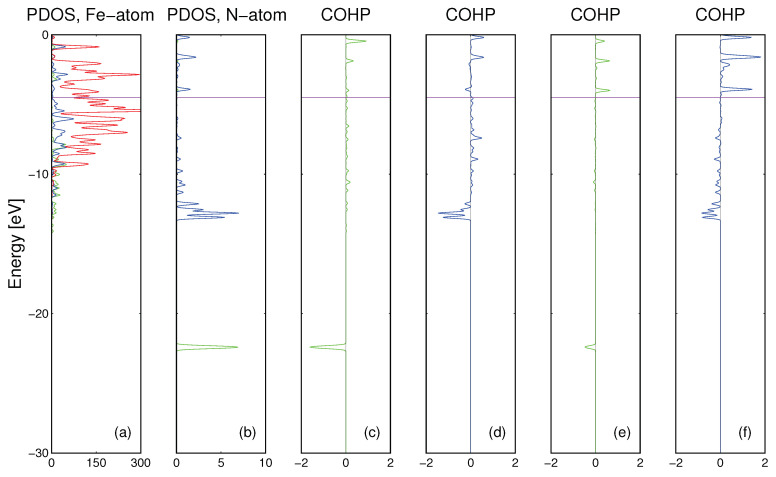
*Ab initio* energy states of the supercell consisting of 108 Fe and a single interstitial N atom: (**a**) PDOS of Fe atoms (green, blue and red line denote *Fe4s*, *Fe4p* and *Fe3d* states, respectively), (**b**) PDOS of N atom (green and blue line denote *N2s* and *N2p* states, respectively) (**c**) COHP of *N2s-Fe4s4p* states, (**d**) COHP of *N2p-Fe4s4p* states, (**e**) COHP of *N2s-Fe3d* states, (**f**) COHP of *N2p-Fe3d* states. The results are obtained for spin-polarized calculations, so the two peaks correspond to different spin orientations. Fermi level is denoted by horizontal magenta line.

The *ab initio* calculation results for nitrogen atom immersed in the Fe matrix are presented in Figure 5. As it is shown the Fe atoms in the matrix are bonded by *Fe3d* orbitals while the N atom could be bonded by its own *N2s* and *N2p* orbitals. The bonding has complex character, the *N2s* overlap with *Fe4s* and *Fe4p* states is bonding while *N2s* states are bonded to *Fe3d* states very weakly. Thus, the bonding of *N2s* states is essentially absent. In contrast to that, the bonding of *N2p* states is strong, to both *Fe4s* and *Fe4p* and to *Fe3d* orbitals. In summary, the interstitial nitrogen atom bonding to the Fe matrix is essentially only via *N2p* states.

Please note that the approach is different from the one applied to the Ga-Fe cluster. The number of Fe atoms is preserved, and a single N atom is added into an interstitial position. Therefore, the energy of dissolution of single nitrogen atom in the Fe matrix is calculated using equation different than Equation (1):

(2) $$\Delta E_{DFT}^{dis}\left(\mathrm{Fe}-N\right)=E_{DFT}\left(108\;\mathrm{Fe}-1\;N\right)-E_{DFT}\left(108\;\mathrm{Fe}\right)-E_{DFT}\left(1\;N\right)$$

Using the calculated DFT values for the right-hand side of the equation, the energy of atomic N dissolution in the Fe matrix is: $\Delta E_{DFT}^{dis}\left(\mathrm{Fe}-N\right)=-5.050$ eV/atom. Noting that the energy of molecular nitrogen is lower by the molecule dissociation contribution $\Delta E_{DFT}^{diss}\left(N{}_{2}\right)=9.801$ eV, the energy of dissolution of single N${}_{2}$ molecule could be obtained as:

(3) $$\Delta E_{DFT}^{dis}\left(\mathrm{Fe}-N{}_{2}\right)=2\Delta E_{DFT}^{dis}\left(\mathrm{Fe}-N\right)+\Delta E_{DFT}^{diss}\left(N{}_{2}\right)$$

Hence the resulting energy of dissolution of molecular nitrogen in Fe is $\Delta E_{DFT}^{dis}\left(\mathrm{Fe}-N{}_{2}\right)=-0.299$ eV/mol. The negative value, indicates a reduction in energy at dissolution of molecular nitrogen, thus promoting high solubility of nitrogen in Fe.

The cluster used for *ab initio* calculations describing N interaction with the Ga matrix is presented in Figure 2b.

**Figure 6:**
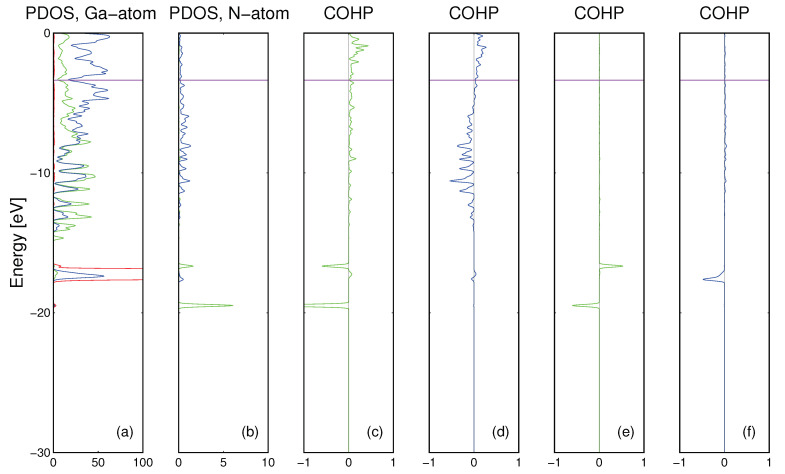
*Ab initio* obtained energy states properties of the supercell consisting of 144 Ga and single N atom: (**a**) PDOS of Ga atoms (green, blue and red line denote *Ga4s*, *Ga4p* and *Ga3d* states, respectively), (**b**) PDOS of N atom (green and blue line denote *N2s* and *N2p* states, respectively,) (**c**) COHP of *N2s-Ga4s4p* states, (**d**) COHP of *N2p-Ga4s4p* states, (**e**) COHP of *N2s-Ga3d* states, (**f**) COHP of *N2p-Ga3d* states. The results are obtained for spin-polarized calculations, so the two peaks correspond to different spin orientations. Fermi level is denoted by horizontal magenta line.

The results of the calculations for nitrogen atom in the Ga matrix are presented in Figure 6. As it was already shown, gallium matrix atoms are bonded by *Ga4s* and *Ga4p* orbitals while N atom could be bonded by its own *N2s* and *N2p* orbitals. The N-Ga bonding is drastically different from the N-Fe case. The *N2s* bond with Ga states is molecular in character with small energy dispersion and small magnitude while *N2p* states are bonded by large dispersion state, i.e., they are extended in real space—Figure 6d. There is a small contribution of *N2p-Ga3d* bonding, due to extended nature of *N2p* state—Figure 6f. The bonding and antibonding overlaps between *N2s* and *Ga3d* states are compensated. Therefore as it is shown above, bonding of interstitial N atom to Fe and Ga matrices has different character.

Please note that the approach is similar to the one applied above to the Fe-N cluster. The number of Ga atoms is preserved, and a single N atom is added. The energy of dissolution of nitrogen single atom in the Fe matrix is calculated using Equation (2). From the obtained DFT values, the energy of atomic N dissolution in the Ga matrix is: $\Delta E_{DFT}^{dis}\left(\mathrm{Ga}-N\right)=4.633$ eV/atom. Then the energy of dissolution of molecular nitrogen is obtained via Equation (3). Thus, the energy of dissolution of molecular nitrogen in Ga is: $\Delta E_{DFT}^{dis}\left(\mathrm{Ga}-N{}_{2}\right)=0.535$ eV/mol. The energy is positive and significantly higher than for Fe, therefore the energy increase should be observed at dissolution of molecular nitrogen, and accordingly, the solubility of nitrogen in liquid Ga should be drastically lower than in Fe.

**Figure 7:**
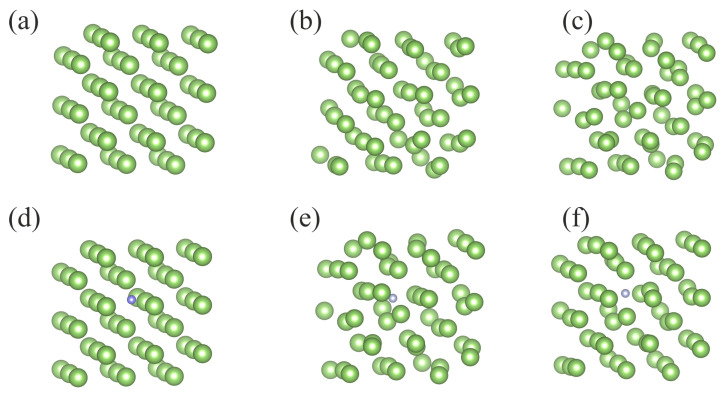
Configuration of Ga atom cluster in MD simulations (**a**–**c**) 54 Ga atoms only, (**d**–**f**) 54 Ga atoms and 1 N atom: (**a**,**d**) initial configuration—0 MD steps, (**b**,**e**) after 10,000 MD steps, (**c**,**f**) after 20,000 MD steps. Green and blue balls denote Ga and N atoms respectively.

It was found however that the obtained total energy of the Ga-N system changes considerably, depending on the configuration used [34]. Therefore, in order to average over configurational degrees of freedom and obtain representation relevant for the liquid, the molecular dynamics (MD) *ab initio* simulations of the 54 Ga atom cluster, with and without a single N atom immersed in, have been performed. In such a model, the corresponding concentration of nitrogen was 0.01851 atomic fraction (at. fr.) The average temperature of the simulation was set to 500 K. The configurations of the system, the initial one and the ones after prescribed number of MD steps, is presented in Figure 7. In fact, the temperature 500 K is above the melting point of Ga ($T_{\mathrm{Ga}}^{M}=303$ K at normal pressure). Therefore, after a short initial period, the configuration of atoms is changed completely. Thus, the initial configuration has no influence on the time averaged data.

**Figure 8:**
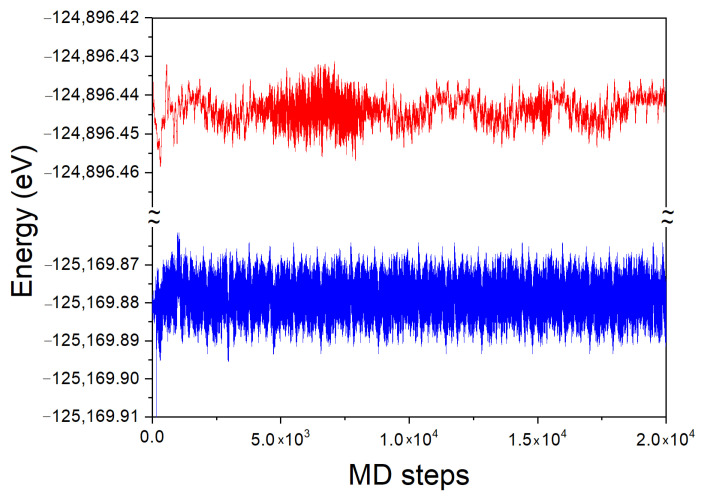
Time evolution of the total energy of 54 Ga atom cluster: red line—without N, blue line—with single N atom inside. The time step was τ = 2 fs = $2\times 10^{-15}$ s. The total simulation time was equal $2\times 10^{4}$ steps = 40 ps = $4\times 10^{-11}$ s.

The time evolution of the total energy of the system for the 54 Ga atom cluster, with and without a single N atom immersed in, is presented in Figure 8. In the simulation, the averaging was made using a sequence of time steps until a steady state has been achieved, as shown in Figure 8. The averaging for the final result was undertaken when the error in the simulation was smaller than 0.001 of the total energy value.

Due to periodic boundary conditions, the system is not closed. Therefore, the energy of the system can change in time. The initial time evolution of the Ga cluster both with and without N atom is similarly fast. The system attains the energy, close to the average one after several hundred MD steps. As it is visible, the energy of the system fluctuates around the average one. The temporal evolution of the fluctuations is different in both cases. The system without N atom exhibits two different fluctuation types: first short lived one, typical for thermal noise of independent thermal motions of single atoms, and the second one which is long lived fluctuations typical for hydrodynamics, i.e., common motion of higher number of atoms. Due to sheer size of the simulation cell, this number cannot be too large. Anyway, such a phenomenon is clearly observed.

The system with the immersed N atom behaves differently. The thermal fluctuations are present with the noise similar to the previous one. Nevertheless, the hydrodynamic fluctuations are absent which may be attributed to the small size of the simulated system. Nitrogen atom strongly binds several atoms in the first coordination zone, hampering their motion. The remaining atoms are not able to move in coordinated manner, thus the system fluctuations are reduced to the thermal noise only.

The time averaged total energy was $E_{DFT}^{MD}$(54 Ga-1 N) = −125,169.879 eV and $E_{DFT}^{MD}\left(54\;\mathrm{Ga}\right)$ = −124,896.443 eV for these two clusters, respectively. The DFT energy of single N atom is $E_{DFT}\left(1\;N\right)=-269.422$ eV. In order to derive the DFT energy of dissolution, it should be noted that the total energy derived from the MD simulations contains two additional terms, not present in the standard DFT calculations. The first one is the average energy of thermal motion of atoms, which for a single nitrogen atom, is $\Delta E_{T}\left(1\;N\right)=3k_{B}T$, the value that arises from both kinetic and potential energy average. The second contribution is the zero-point energy $E_{l}^{ZPE}$(N-Ga). For the above simulations conducted at *T* = 500 K, the thermal energy value is $\Delta E_{T}\left(1\;N\right)=0.129$ eV. From the standard DFT calculations at *T* = 0 K, the zero-point energy value is $E_{l}^{ZPE}\left(N-\mathrm{Ga}\right)=0.204$ eV. Thus the N atom dissolution energy is given by relation different from Equation (2):

(4) $$\Delta E_{DFT}^{dis}\left(\mathrm{Ga}-N\right)=E_{DFT}^{MD}\left(54\;\mathrm{Ga}-1\;N\right)-E_{DFT}^{MD}\left(54\;\mathrm{Ga}\right)-E_{DFT}\left(1\;N\right)-\Delta E_{T}\left(1\;N\right)-E_{l}^{ZPE}\left(N-\mathrm{Ga}\right)$$

From the above data, the DFT energy of dissolution of N in liquid Ga is $\Delta E_{DFT}^{dis}\left(\mathrm{Ga}-N\right)=-4.347$ eV/atom. As mentioned earlier, the dissolution energy of molecular nitrogen should be lowered by the energy of N${}_{2}$ molecule dissociation $\Delta E_{DFT}^{diss}\left(N{}_{2}\right)=9.801$ eV. Accordingly, the energy of dissolution of a single N${}_{2}$ molecule in Ga is given by:

(5) $$\Delta E_{DFT}^{dis}\left(\mathrm{Ga}-N{}_{2}\right)=2\Delta E_{DFT}^{dis}\left(\mathrm{Ga}-N\right)+\Delta E_{DFT}^{diss}\left(N{}_{2}\right)$$

Therefore, the resulting energy of dissolution of molecular nitrogen in Ga(l): $\Delta E_{DFT}^{dis}\left(\mathrm{Ga}-N{}_{2}\right)=1.107$ eV/mol. The energy change associated with single atom is half of the one for molecule, i.e., $\Delta E_{DFT}^{dis}\left(\mathrm{Ga}-N{}_{2}\right)=0.553$ eV/atom. The energy is positive, therefore the energy increase occurs at dissolution of molecular nitrogen that leads generally, to relatively low N solubility in Ga as was already observed [11,35,36].

**Figure 9:**
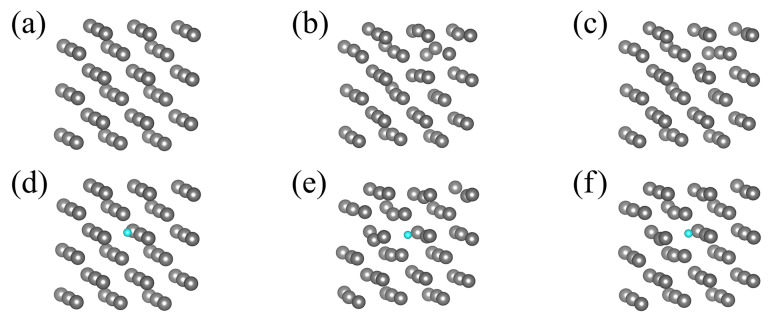
Configuration of Fe atom cluster in MD simulations (**a**–**c**) 54 Fe atoms only, (**d**–**f**) 54 Fe atoms and 1 N atom: (**a**,**d**) initial configuration—0 MD steps, (**b**,**e**) after 10,000 MD steps, (**c**,**f**) after 20,000 MD steps. Gray and cyan balls denote Fe and N atoms respectively.

Similar MD simulations were made for N dissolution in Fe using 54 Fe atoms cluster with single N atom immersed in the metal as presented in Figure 9. As above, the concentration of nitrogen was 0.01851 at. fr. The technical details of the simulations were the same as these used for Ga and the temperature was again set at 500 K.

The evolution of the system looks differently than for the Ga case. At 500 K, Fe is in the solid phase, therefore the evolution of the system is typical for the crystal. The configurations shown in both Fe and Fe:N cases are typical for the solid phase where the long-range order is preserved, and the atoms are attached to their lattice sites. The presence of nitrogen does not induce melting, the order is still preserved. Nevertheless, the fluctuations change the lattice in a visible manner.

**Figure 10:**
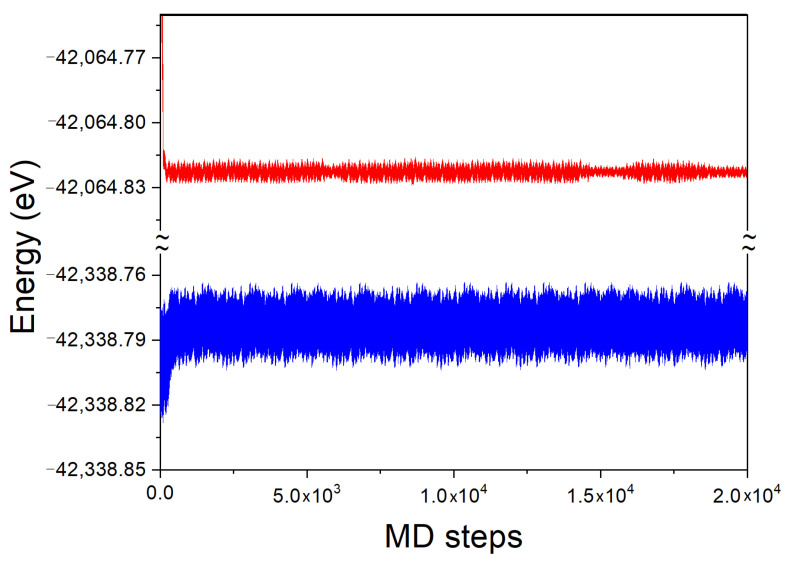
Time evolution of the total energy of 54 Fe atom cluster: red line—without N atom, blue line—with single N atom. The time step and the simulation time are identical as those in Figure 8.

The time evolution of the system total energy for the Fe and Fe-N clusters, are presented in Figure 10. As before, the final result was undertaken when the error in the simulation was smaller than 0.001 of the total energy value.

As above, due to periodic boundary conditions, the system is not closed so that the energy of the system may change in time. The time evolution of the Fe cluster both with and without N atom is the same as for the Ga case, similarly fast. The system attains the energy, close to average one after several hundred MD steps. The energy of the system fluctuates around the average. The thermal fluctuations are present with the noise similar but larger for N atom present. The hydrodynamic fluctuations are absent as the system is essentially solid.

The time averaged total energy was $E_{DFT}^{MD}\left(54\;\mathrm{Fe}-1\;N\right)$ = −42,338.783 eV and $E_{DFT}^{MD}\left(54\;\mathrm{Fe}\right)$ = −42,064.823 eV for these two clusters, respectively. As for Ga cluster, the DFT energy of single N atom and the kinetic energy must be taken into account, using the same values, i.e., $E_{DFT}\left(1\;N\right)$ = −269.422 eV and $\Delta E_{T}\left(1\;N\right)=0.129$ eV, respectively. The zero-point energy value from standard DFT calculations is $E_{l}^{ZPE}\left(N-\mathrm{Fe}\right)=0.210$ eV. The N atom dissolution energy, obtained from Equation (4) is $\Delta E_{DFT}^{dis}\left(\mathrm{Fe}-N\right)=-4.867$ eV/atom. The energy of dissolution of single N${}_{2}$ molecule in Fe, given by Equation (5) is $\Delta E_{DFT}^{dis}\left(\mathrm{Fe}-N{}_{2}\right)=0.003$ eV/mol, and accordingly the part associated with single atom is: $\Delta E_{DFT}^{dis}\left(\mathrm{Fe}-N{}_{2}\right)=0.001$ eV/atom. The energy is almost zero, i.e., much lower than the value obtained for liquid Ga, indicating that the solubility of nitrogen in Fe should be much higher at the otherwise, similar conditions.

### 3.3. Equilibrium between N${}_{2}$ Gas and Nitrogen Dissolved in Ga and Fe Metals

The equilibrium between gas and liquid phases entails equality of chemical potentials. In case of dissolution of nitrogen from the diatomic form in its gas phase in a metallic liquid it is recognized that the N${}_{2}$ molecule dissociates in contact with liquid metals. For Ga solvent, it was demonstrated by Romanowski et al. [12,13] by DFT modeling of both N${}_{2}$ dissociative chemisorption and dissolution processes. As iron has higher bonding energy to nitrogen than gallium, it is expected that dissolution of nitrogen in liquid Fe is also dissociative that leads to universal relation for equilibrium:

(6) $$\frac{1}{2}\mu_{N_{2}\left(v\right)}\left(p,T\right)=\mu_{N(l)}\left(T\right)$$

Direct comparison of the chemical potential of both species is not possible as there is no direct prescription to obtain chemical potential at given temperature and pressure.

The chemical potential equality can be analyzed by calculating the enthalpy and entropy differences for nitrogen in the liquid and gas phases separately and combine them in the Gibbs free energy. That causes considerable difficulties as in the standard approach, the enthalpy difference at zero temperature, equal to the *ab initio* energy difference between the vapor and the liquid, is considered. The other contributions are treated as entropy term, i.e., their contribution to chemical potential is proportional to the temperature. Typically, the value of the proportionality constant is adjusted by fit to the available experimental data.

The entropy difference could not be obtained at zero K as the vapor-phase entropy has singularity there (for discussion of the singularity please refer to Appendix A). Therefore, the separate calculation path was proposed for the total difference in chemical potential at the condensed/vapor, i.e., liquid (or solid)/vapor (l-v) phase transition for nitrogen. The enthalpy difference at the solid-vapor transition is calculated at zero K. The entropy difference at this transition is obtained at normal conditions. The difference in total chemical potential must be supplemented by changes occurring during the transition to these points. The entire paths are fairly complex, nevertheless, the same results were obtained independently by Jackson and Walsh [37] for bulk and by us for the properties of gas adsorbed at the surfaces of solids [38]. The equations here are written in formulation given in reference [38], with the sign reversed, i.e., using dissolution energy as defined in Section 2.1, for the vapor–solid chemical potential difference:

(7) $$\begin{array}{c} \begin{array}{cc} \Delta \mu_{lv}\left(p,T\right) & =\mu_{N(l)}\left(T,x\right)-\frac{1}{2}\mu_{N_{2}\left(v\right)}\\ \; & =\Delta H_{dis}^{DFT}\left(0\right)+\Delta G_{S-dis}+\Delta H_{therm}+\Delta G_{S-therm}+\Delta G_{pres}+\Delta G_{diss}\left(T,x\right)\\ \; & =\Delta H_{dis}^{DFT}-T_{0}\Delta s_{lv}+\int_{0}^{T}\left(C_{l}-C_{v}\right)dT-\int_{T_{0}}^{T}\left(s_{l}-s_{v}\right)dT+\int_{p_{0}}^{p}\left(v_{l}-v_{v}\right)dp+k_{B}Tln\left(x\right) \end{array} \end{array}$$

where the normal temperature and pressure are defined as: *T*${}_{0}$ = 20 °C = 298.15 K and *p*${}_{0}$ = 1 bar. The terms in the above equation are defined as:

(i) The first term is the enthalpy change at dissolution for single N atom, calculated as *ab initio* energy difference between the vapor and the solid (liquid):
  (8) $$\Delta H_{dis}^{DFT}\left(0\right)=h_{l}\left(0\right)-h_{v}\left(0\right)$$
  It was shown recently [39] that the energy difference obtained from DFT calculations (Equation (1)) does not correspond to the enthalpy change during vaporization as the thermodynamic state energy is increased by the energy of vibrations at the ground state, the effect called zero-point energy (ZPE) [39]. As these values are not identical, they contribute to the enthalpy difference giving
  (9) $$\Delta H_{dis}=\Delta E_{dis}^{DFT}+\Delta E_{lv}^{ZPE}$$
  where the zero-point energy difference is:
  (10) $$\Delta E_{lv}^{ZPE}=E_{l}^{ZPE}-E_{v}^{ZPE}$$
  As pointed out by Ponomareva et al. [34] and discussed in Section 2.1, the energy depends on the atomic configuration used for the calculation. Therefore, in our approach, the enthalpy difference was evaluated for configurations resulting from the MD simulations at 500 K. As discussed in Section 2.1, the dissolution energy was obtained using its MD value, the average thermal energy of single N atom at 500 K $\Delta E_{T}$(1 N) and zero-point energy $E_{l}^{ZPE}$(N-Ga) (see Equation (4)).
(ii) Difference in chemical potential related to the entropy change at dissolution, calculated at normal conditions,
  (11) $$\Delta G_{S-dis}=-T_{0}\Delta s_{lv}=-T_{0}\left(s_{l}-s_{v}\right)$$
(iii) Difference in enthalpy change between both phases at the transition from 0 K to normal conditions, i.e., $T_{0}$,
  (12) $$\begin{array}{cc} \Delta H_{therm}= & \left[H_{l}\left(T_{0}\right)-H_{v}\left(T_{0}\right)\right]-\left[H_{l}\left(0\right)-H_{v}\left(0\right)\right]\\ = & \left[H_{l}\left(T_{0}\right)-H_{l}\left(0\right)\right]-\left[H_{v}\left(T_{0}\right)-H_{v}\left(0\right)\right]=\int_{0}^{T_{0}}\left(C_{l}-C_{v}\right)dT \end{array}$$
(iv) The chemical potential change caused by the temperature change from $T_{0}$ to,
  (13) $$\begin{array}{cc} \Delta G_{S-therm}= & \left[G_{l}\left(T\right)-G_{v}\left(T\right)\right]-\left[G_{v}\left(T_{0}\right)-G_{v}\left(T_{0}\right)\right]\\ = & \left[G_{l}\left(T\right)-G_{l}\left(T_{0}\right)\right]-\left[G_{v}\left(T\right)-G_{v}\left(T_{0}\right)\right]=-\int_{T_{0}}^{T}\left(s_{l}-s_{v}\right)dT \end{array}$$
(v) The pressure induced change of the chemical potential
  (14) $$\Delta G_{pres}=\int_{p_{0}}^{p}\left(v_{l}-v_{v}\right)dp-k_{B}Tlna+\int_{p_{0}}^{p}v_{l}dp≅-k_{B}Tln\left(\frac{p}{p_{0}}\right)$$
  as $v_{v}>>v_{l}$.
(vi) Term related to the concentration of N in the metal liquid. The latter contribution may be calculated using standard chemical approximations for ideal solutions:
  (15) $$\Delta G_{diss}\left(T,x\right)=k_{B}Tln\left(x\right)$$
  where *x* is the concentration of N atoms in the solution.

### 3.4. Dissolution of Molecular Nitrogen in Liquid Gallium

The enthalpy of dissolution ((i) in the previous Section) for a single N atom consists of two contributions: energy of dissolution $\Delta E_{dis}^{DFT}\left(\mathrm{Ga}-\frac{1}{2}N{}_{2}\right)=\frac{\Delta E_{DFT}^{dis}\left(\mathrm{Ga}-N{}_{2}\right)}{2}=0.553$ eV/atom, and the zero-point energy contribution, given in Equation (10), calculated as the difference of the zero-point energies for single N atom in the liquid $E_{l}^{ZPE}\left(N-\mathrm{Ga}\right)=0.204$ eV and that derived from vibrations in the N${}_{2}$ molecule in the vapor $E_{v}^{ZPE}\left(\frac{1}{2}N{}_{2}\right)=\frac{E_{v}^{ZPE}\left(N{}_{2}\right)}{2}=0.0712$ eV. Therefore, zero-point energy difference is $\Delta E_{lv}^{ZPE}\left(\mathrm{Ga}-\frac{1}{2}N{}_{2}\right)=0.143$ eV.

In summary, the enthalpy of dissolution for single N atom in the gallium metal, from N${}_{2}$ molecule is: $\Delta H_{dis}\left(\mathrm{Ga}-\frac{1}{2}N{}_{2}\right)=0.696$ eV/atom.

Please note that the contribution of dissolution entropy to the free energy (ii) $\Delta G_{S-vap}\left(\mathrm{Ga}-\frac{1}{2}N{}_{2}\right)$ could be easily obtained. As both the vapor and the liquid N-containing phases are not ordered, the entropy change could be expressed by the appropriate volume ratio:

(16) $$\Delta G_{S-dis}=-T_{0}\Delta s_{lv}=-k_{B}T_{0}ln\left(\frac{v_{l}}{v_{v}}\right)$$

From the ideal gas law, the volume associated with single N atom at normal temperature and pressure is: $v_{v}\left(N\right)=\frac{v_{v}\left(N{}_{2}\right)}{2}=1.861\times 10^{4}$ Å${}^{3}$. The volume associated with incorporation of the N atom into the Ga cluster is $v_{l}\left(N\right)=2.990$ Å${}^{3}$. Thus, the dissolution entropy related free energy change described by Equation (16) is: $\Delta G_{S-vap}\left(\mathrm{Ga}-\frac{1}{2}N{}_{2}\right)=0.224$ eV.

For the vapor phase, the entropy terms could be evaluated quite easily. In a good approximation, the specific heat of nitrogen in the gas phase is determined by the equipartition principle, i.e., it is constant. The thermal contributions (iii) and (iv) could be obtained be separate calculation for both phases. The vapor-phase contribution is obtained directly, as the heat capacity is constant.

The enthalpy difference is $\Delta H_{therm}\left(N{}_{2}\right)=H_{v}\left(T_{0}\right)-H_{v}\left(0\right)=8.67\;\mathrm{kJ}/\mathrm{mole}=8.986\times 10^{-2}$ eV/molecule. The latter value for single N${}_{2}$ molecule could be recalculated for single N atom as: $\Delta H_{therm}\left(\frac{1}{2}N{}_{2}\right)=H_{v}\left(T_{0}\right)-H_{v}\left(0\right)=4.493\times 10^{-2}$ eV/atom. Generally, the contribution $\Delta H_{therm}\left(N{}_{2}\right)$ is small in relation to the enthalpy change at dissolution or to the difference in chemical potential related to dissolution entropy change thus not affecting the entire result considerably.

The second term is the nitrogen free energy difference between standard *T*${}_{0}$ and a selected temperature *T*. This could be directly obtained from already published data concerning the chemical potential of gaseous nitrogen at normal pressure [40]:

(17) $$\mu_{N}^{0}\left(y\right)=\frac{\mu_{N_{2}}^{0}\left(T\right)}{2}=-4.86-0.967y-0.1013y^{2}+0.0173y^{3}$$

where $y\equiv \frac{T}{1000}$ is scaled temperature. From this data the free energy difference is readily obtained as:

(18) $$\Delta G_{S-therm}\left(\frac{1}{2}N{}_{2}\right)=G_{v}\left(T\right)-G_{v}\left(T_{0}\right)=1.156-0.967y-0.1013y^{2}+0.0173y^{3}$$

The entropy contribution in the liquid may be obtained from evaluation of phonon related effects, directly. The phonon part of the vibrational energy $E^{vib}$, the specific heat $C^{vib}$, entropy $S^{vib}$, and free energy $F^{vib}$ associated with the nitrogen atom immersed in gallium may be obtained as [39,41]:

(19)Evib(x)=kBT∑jxjexp(xj)−1,(20)Cvib(x)=kB∑jxj2exp(xj)[exp(xj)−1]2,(21)Svib(x)=kB∑jxjexp(xj)−1−ln[1−exp(−xj)](22)Fvib(x)=kBT∑jln[1−exp(xj)]

where $x_{j}\equiv \frac{\hbar \omega_{j}}{k_{B}T}$ and $\omega_{j}$ is phonon frequency of a *j-th* phonon mode. A simplified treatment may be used, based on Debye theory in which the acoustic phonon frequencies are approximated by linear dependence. Accordingly, the maximum phonon energy, known as the Debye energy, and its equivalents: frequency and the temperature, are related as follows: $E_{D}=\hbar \omega {}_{D}=k_{B}\theta {}_{D}$. Using this simplified representation, the spectra sums are replaced by integrals, giving:

(23)Evib(x)=9kBT(TθD)3∫0θD/Tx3dxexp(x)−1,(24)Cvib(x)=9kB(TθD)2∫0θD/Tx4exp(x)dx[exp(x)−1]2,(25)Svib(x)=9kB(TθD)3∫0θD/T{xexp(x)−1−ln[1−exp(−x)]}x2dx(26)Fvib(x)=9kBT(TθD)3∫0θD/Tx2ln[1−exp(−x)]dx

The N-Ga Debye temperature was obtained from the fit of *ab initio* derived specific heat to the temperature dependence given by Equations (23)–(26). The obtained value equal $\theta_{D}=552$ K was used to evaluate other thermal contributions given by Equations (23)–(26).

Evaluating the pressure related term ((v) in the previous Section) one must account that in Equation (17), the pressure of molecular nitrogen is used. In our model, the expression scaled to atomic nitrogen has to be used, i.e., $\Delta G_{pres}\left(N\right)=\frac{1}{2}\Delta G_{pres}\left(N{}_{2}\right)=-\frac{k_{B}T}{2}ln(\frac{p}{p_{0}})$, where *p*${}_{0}$ = 1 bar.

At equilibrium, the chemical potential difference vanishes, i.e., $\Delta \mu {}_{vl}\left(p,T\right)=0$ which allows us to express the equilibrium pressure above specified liquid phase via chemical potential contribution in the ideal gas approximation:

(27) $$\begin{array}{c} \begin{array}{cc} \frac{k_{B}T}{2}ln\left(a\right) & ≅\frac{k_{B}T}{2}ln(\frac{p}{p_{0}})=\\ \; & =\Delta H_{dis}+\Delta H_{therm}+\Delta G_{S-therm}+\Delta G_{pres}+\Delta G_{diss}\left(T,x\right)=\\ \; & =-\Delta E_{des}^{DFT}-\Delta E_{vl}^{ZPE}+T_{0}\Delta s_{vl}-\int_{0}^{T}\left(C_{v}-C_{l}\right)dT+\int_{\left(T_{0}\right)}^{T}\left(s_{l}-s_{v}\right)dT+k_{B}Tln\left(C_{N}\right) \end{array} \end{array}$$

In the real gas case (at high pressure), the pressure *p* in Equation (27) must be interpreted as fugacity of N${}_{2}$ gas (or activity, if divided by *p*${}_{0}$) accounting for intermolecular interactions due to high density of the gas.

**Figure 11:**
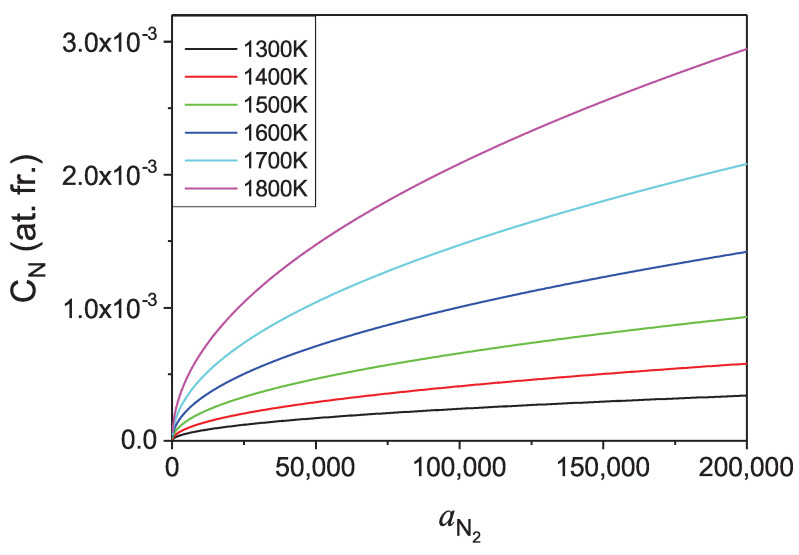
Solubility of nitrogen in liquid gallium in function of molecular nitrogen activity, for several temperatures.

Therefore, the dependence of the concentration of nitrogen in liquid Ga on activity of the N${}_{2}$ gas over the solution could be obtained. Such a dependence for several temperatures is presented in Figure 11.

**Figure 12:**
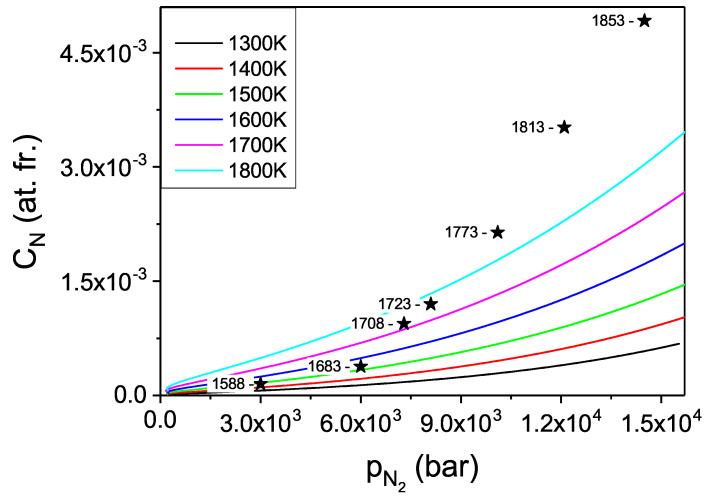
Solubility of nitrogen in liquid gallium in function of molecular nitrogen pressure for several selected temperatures. The available experimental data (black stars) [35] are included for comparison.

As in expressions for thermodynamic functions, the considered activities of N${}_{2}$ gas replace the pressure where ideal gas equation of state is not fulfilled, the corresponding pressure had to be evaluated using the modified equation of state. Such equation of state of nitrogen derived from experimental data by Jacobsen et al. [42] and confirmed by molecular dynamics and *ab initio* simulations by Strak et al. [43,44] was used for the activity to pressure transition. In such a way, the nitrogen solubility as a function of pressure of N${}_{2}$ pressure was evaluated and is presented in Figure 12.

From the presented calculations, it follows that the solubility of nitrogen in liquid Ga is low, and accordingly, high pressures are required to attain technically viable rates of GaN crystallization [11,35,36]. The technically used N${}_{2}$ pressure was recovered using nitrogen equation of state for high pressures and high temperatures. It is interesting to mention that thermodynamic activity of a compressed N${}_{2}$ gas at technically relevant pressures of the order of 10 kbar, is nominally much higher than the corresponding pressure itself. For example at 10 kbar of the molecular nitrogen pressure, the N${}_{2}$ activity is about $a_{N{}_{2}}≅141,900$ at *T* = 1300 K, i.e., about 14 times higher than the ideal gas value.

The solubility data [34] experimentally evaluated for conditions corresponding to *p-T* coordinates of Ga-GaN-N${}_{2}$ triple points are in reasonably good agreement with the presented *ab initio* results. The result is impressive as the obtained concentrations are exponentially dependent on the calculated quantities, i.e., thermodynamic potentials. The agreement is much better for lower concentrations where the ideal solution approximation is more precise. For higher pressures, the calculated data are consistently smaller than the measured ones, which is attributed to deviation from ideal solution approximation.

The calculated concentrations are relatively small, in the range of several atomic promilles, as already determined experimentally. They limit the possible crystallization rates as they are proportional to the equilibrium concentration and the supersaturation. The latter could be established using temperature difference, i.e., the difference in solubility, normalized to the equilibrium concentration. Thus, the temperature dependence is of importance here. As it is shown in Figure 12, the concentration changes rapidly with the temperature, allowing the obtaining of high supersaturation via temperature difference between the dissolution and the growth zone in the metal. Unfortunately, high supersaturation accelerated growth often leads to pronounced Mullins-Sekerka instability [45,46] and consequently deterioration of the quality of the resulting crystals [47].

**Figure 13:**
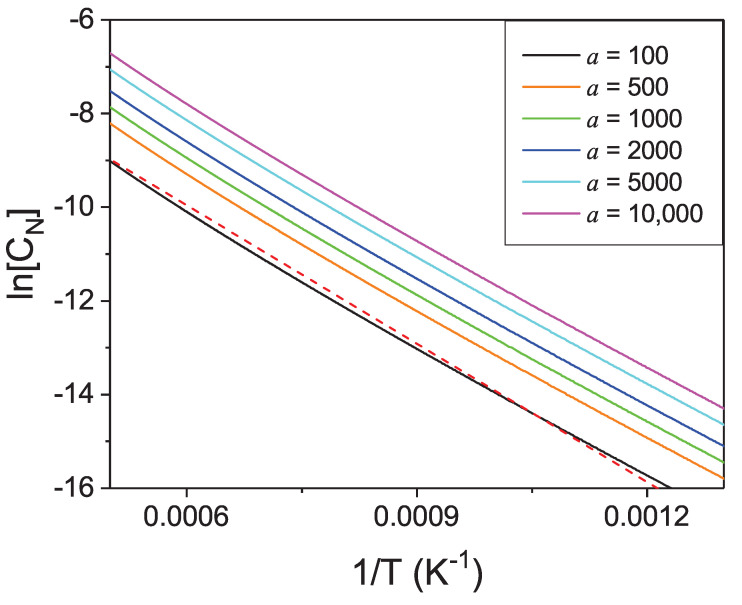
Logarithm of solubility of nitrogen in liquid gallium in function of the inverse of the temperature for several values of activity of molecular nitrogen. The red line represents linear fit to the low pressure line.

From this point it is interesting to analyze the temperature dependence of nitrogen solubility at constant activity, shown in Figure 13. These data could be used in the plot of the Van’t Hoff type following the Van’t Hoff relation:

(28) $$C_{N}\left(T\right)=exp[-\frac{\Delta H_{therm}^{dis}}{k_{B}T}+\frac{\Delta s_{therm}^{dis}}{k_{B}}]$$

to determine thermodynamic heat of dissolution $\Delta H_{therm}^{dis}$.

From the linear fit, the value: $\Delta H_{therm}^{dis}=0.848\pm 0.001$ eV/atom was obtained what is far from the *ab initio* obtained value $\Delta H_{dis}\left(\mathrm{Ga}-\frac{1}{2}N{}_{2}\right)=0.696$ eV/atom. As it is shown, the plot deviates from linear regime significantly. Such dependence is not typical for enthalpy dominated phase transition as the entropy related change of the enthalpy of dissolution is about 21% for the temperature increase to 1800 K. That confirms importance of the entropy related terms in the dissolution of nitrogen in liquid Ga, indicating that the above combined energy-entropy approach is necessary to obtain good agreement with the experimental data.

### 3.5. Dissolution of Molecular Nitrogen in Metallic Iron

Similar analysis could be made for dissolution of N-in-Fe. The enthalpy of vaporization (i) (in Section 3.3) for single N atom from Fe solution consists of the two contributions. First is the energy of dissolution, equal to $\Delta E_{dis}^{DFT}\left(\mathrm{Fe}-\frac{1}{2}N{}_{2}\right)=\frac{\Delta E_{DFT}^{dis}\left(\mathrm{Fe}-N{}_{2}\right)}{2}=0.001$ eV/atom. Its value favors dissolution of nitrogen in metallic Fe. The second is zero-point energy difference, in the solution $E_{l}^{ZPE}\left(N-\mathrm{Fe}\right)=0.210$ eV, and already used molecular value in the vapor $E_{v}^{ZPE}\left(\frac{1}{2}N{}_{2}\right)=\frac{E_{l}^{ZPE}\left(N{}_{2}\right)}{2}=0.0712$ eV, i.e., equal to $\Delta E_{lv}^{ZPE}\left(\mathrm{Fe}-\frac{1}{2}N{}_{2}\right)=0.139$ eV. Altogether, the enthalpy of dissolution is $\Delta H_{dis}\left(\mathrm{Fe}-\frac{1}{2}N{}_{2}\right)=0.172$ eV.

The entropy of dissolution may be obtained from *ab initio* data. The volume associated with N atom in the Fe cluster is $v_{l}\left(N\right)=2.200$ Å${}^{3}$. Using the ideal gas volume for normal pressure and temperature $v_{v}\left(N\right)=\frac{v_{v}\left(N{}_{2}\right)}{2}=1.861\times 10^{4}$ Å${}^{3}$ the dissolution free energy change could be obtained from Equation (5) to get $\Delta G_{S-vap}\left(\mathrm{Fe}-\frac{1}{2}N{}_{2}\right)=0.232$ eV. Thus, this value is only slightly different from that obtained for liquid Ga.

The entropy terms could be obtained following these for the previous case. The enthalpy difference for vapor is identical and equal to: $\Delta H_{therm}\left(\frac{1}{2}N{}_{2}\right)=H_{v}\left(T_{0}\right)-H_{v}\left(0\right)=3.251\times 10^{-2}\;\mathrm{eV}/\mathrm{atom}$. The free energy difference of gaseous nitrogen is given by Equation (7). The remaining solid contributions may be obtained from Equation (16), using the Debye temperature which for N dissolved in metallic Fe was equal $\theta {}_{D}=512$ K.

**Figure 14:**
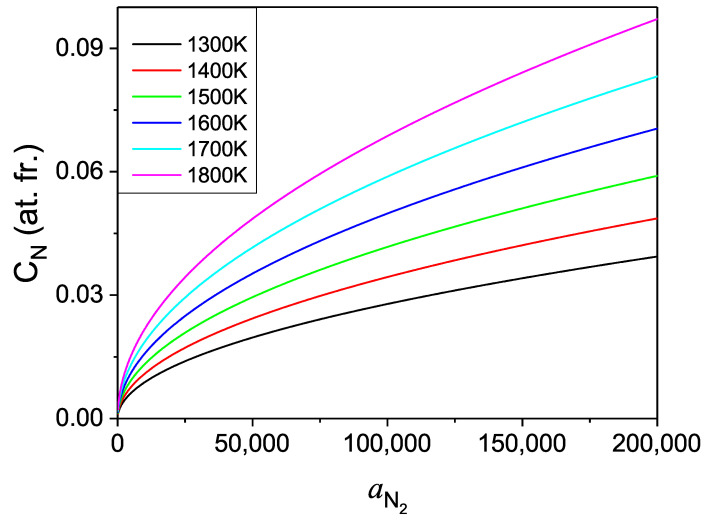
Solubility of nitrogen in liquid Fe in function of molecular nitrogen activity for several temperatures.

The pressure dependence of the N concentration in the Fe solvent could be obtained. Such data are presented in Figure 14. Please note that in the presented diagrams the pressure related coordinate represents the chemical potential of compressed N${}_{2}$ gas, i.e., the activity. The explicit pressure dependence should be recovered expressing the pressure as a function of activity via nitrogen equation of state.

**Figure 15:**
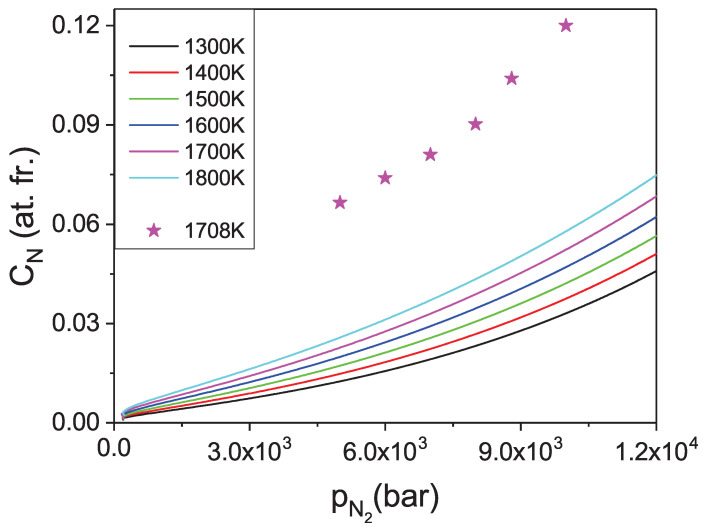
Solubility of nitrogen in liquid Fe in function of molecular nitrogen pressure for several temperatures. The stars represent experimental data obtained at T = 1708 K.

From the obtained data it follows that the concentration values are more than one order of magnitude higher than in case of gallium. Thus, the Fe-based solution seems to be a much better option for efficient crystallization of large GaN single crystals. More physical insight may be obtained using technically relevant variable i.e., the pressure, which is recalculated from activity using the equation of state [43,44,45]. The corresponding results are presented in Figure 15. The experimental solubility data obtained according to the procedure described in Section 2.2, for a constant temperature of 1708 K, are also included into the diagram. The experiment clearly confirmed high concentration of N in the Fe metal. Also, the character of the isothermal pressure dependence is well reproduced.

The obtained theoretical solubility is approximately half of the measured values. This is most likely related to the two factors: first mentioned in Ga case, is related to nonideality of the solution. The effective interaction is much stronger in the case of Fe than in the case of Ga. Therefore the interaction related deviation from ideality is larger. Second factor is related to the enthalpy calculations. In the above *ab initio* modeling only single N atom was inserted in the Fe cluster. In fact in the concentration range of 10 at.%, additional nitrogen atoms may be present in the neighborhood. The N-N attraction is strong, which leads to the decrease of the system energy and consequently lowering its chemical potential thus increasing the nitrogen concentration as observed experimentally.

The theoretical evaluations indicate that the solubility of nitrogen at technically possible limit conditions for large volume high temperature gas reactors, i.e., 10 kbar attains considerable values of about 5.8 at.% at *T* = 1800 K. These data are only slightly reduced by the lower temperatures, i.e., about 3.0 at.% at *T* = 1300 K. Thus, the temperature increase has drastically smaller influence on the concentration of N in iron than in gallium. This can complicate establishing supersaturation in the growth solution necessary for crystallization by application of the temperature gradient.

**Figure 16:**
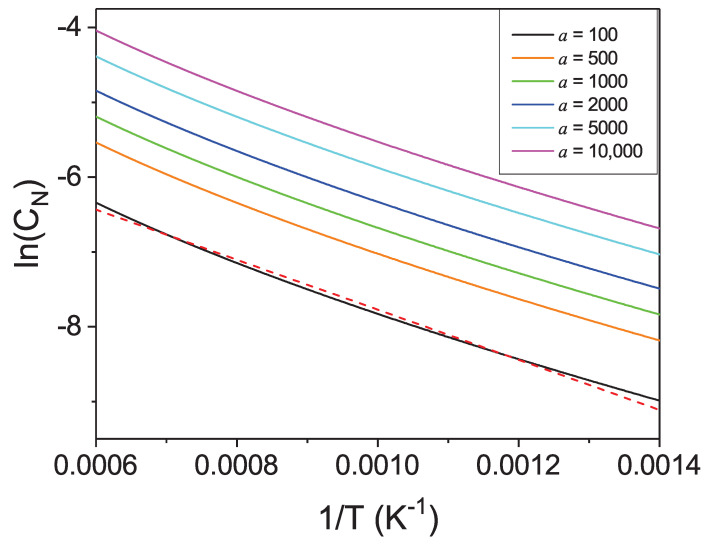
Logarithm of solubility of nitrogen in liquid iron as a function of the inverse of the temperature for several values of pressure of molecular nitrogen (units: [bar]). The red line represent linear fit to the low pressure line.

The temperature dependence may be studied using concentration vs inverse temperature plot for selected values of nitrogen activity as shown in Figure 16. The Van’t Hoff relation (Equation (28)) may be again used to determine thermodynamic heat of dissolution $\Delta H_{therm}^{dis}$. From the linear fit the following value was obtained $\Delta H_{therm}^{dis}=0.2889\pm 0.001$ eV. This is much smaller than the *ab initio* obtained value $\Delta H_{dis}\left(Fe-\frac{1}{2}N{}_{2}\right)=0.172$ eV that indicates that the entropy related, temperature-dependent term drastically lowers the enthalpy of dissolution. Additionally, the solubility does not follow the linear plot of the Van’t Hoff type, similarly to gallium. Even though the interaction term is approximately four times smaller than for gallium, the deviation from linearity is similar that confirms the role of entropy related terms in the dissolution of nitrogen in liquid metals.

The observed temperature dependence poses more stringent requirement for design of crystal growth apparatus in case of Fe solvent. In order to obtain a comparable supersaturation, much larger temperature difference than for gallium, should be applied. This could be beneficial as the best conditions for the growth is small supersaturation. In perspective, large high-quality crystals of GaN could be obtained from solutions in liquid Fe.

## 4. Summary

Dissolution of molecular nitrogen in gallium and iron metals was studied by high-precision *ab initio* calculations. It was shown that N${}_{2}$ is strongly attracted by both metals which led to disintegration of the molecules and dissolution in atomic form. The details of interactions of N atoms with both Ga and Fe metal solvent matrices were revealed by standard *ab initio* calculations at zero K. Nitrogen atoms do not form bonding between their *N2s* states and the surrounding metal atoms. On the contrary, the *N2p* states are involved in bonding in both cases by overlapping with the states of neighboring metal atoms. These metal states are different for Ga and Fe, in case of gallium the bonds are created to Ga4s and Ga4p states while in case of Fe bonds are formed to all states, with the largest overlap to the *Fe3d* ones.

Accordingly, the interaction energies between nitrogen and the metals are different. Direct *ab initio* calculations gave for atomic N dissolution in Fe matrix $\Delta E_{DFT}^{dis}\left(\mathrm{Fe}-N\right)=-5.050$ eV/atom while for Ga matrix that was $\Delta E_{DFT}^{dis}\left(\mathrm{Ga}-N\right)=4.633$ eV/atom. Accounting for high dissociation energy of N${}_{2}$ molecule $\Delta E_{DFT}^{diss}\left(N{}_{2}\right)=9.801$ eV, the N${}_{2}$ dissolution energy in Fe: $\Delta E_{DFT}^{dis}\left(\mathrm{Fe}-N{}_{2}\right)=-0.299$ eV/mol and in Ga $\Delta E_{DFT}^{dis}\left(\mathrm{Ga}-N{}_{2}\right)=0.535$ eV/mol were evaluated. These results indicate that dissolution of nitrogen is energetically much more favorable for Fe than for Ga as solvent.

*Ab initio* MD simulations were also used to determine the energy change during dissolution. The values differ from the direct *ab initio* ones as they include the zero-point energies and the kinetic energy. Nevertheless, the molecular nitrogen dissolution energy values, as in the previous case, are different for both metals: for Ga it is $\Delta E_{DFT}^{dis}\left(\mathrm{Ga}-N{}_{2}\right)=1.107$ eV/mol while for Fe $\Delta E_{DFT}^{dis}\left(\mathrm{Fe}-N{}_{2}\right)=0.003$ eV⁄mol. The difference is large, close to 1 eV, indicating more energy favored dissolution in iron.

Temperature-dependent contributions are large, drastically changing the chemical potential balance between the vapor and the condensed phases. As nitrogen is tightly bound to surrounding metal atoms, the dominant kinetic contribution in the solution stems from N atom vibrations in the cage of the metal environment. As shown in the Appendix A, the ideal gas contribution to entropy is singular at zero K. Therefore the entropy difference is calculated via normal conditions while enthalpy difference is calculated via zero K. The temperature-dependent contribution was obtained from Debye theory, for which the Debye temperature for Ga was $\theta {}_{D}=552$ K and for Fe $\theta {}_{D}=512$ K, i.e., similar values. The Debye contributions are large and temperature-dependent (Equation (13)). For N in liquid Ga, they are $\Delta G_{S-therm}=-0.488$ eV and $\Delta G_{S-therm}=-1.039$ eV for *T* = 1000 K and *T* = 2000 K, respectively. For N-in-Fe, they are $\Delta G_{S-therm}=-0.475$ eV and $\Delta G_{S-therm}=-1.006$ eV for *T* = 1000 K and *T* = 2000 K, respectively. Thus, they are significant and similar for both metals, seriously affecting the equilibrium. The other contributions, such as thermal enthalpy difference (Equation (12)) or concentration dependent are much smaller.

The obtained equilibria for the N${}_{2}$-Ga and N${}_{2}$-Fe systems are much different. The N solubility was obtained for technically amenable part, i.e., for temperatures between 1300 K and 1800 K. The N${}_{2}$ activity was limited to $a\left(N{}_{2}\right)\leq 2\times 10^{5}$ (corresponding to the N${}_{2}$ pressure of 10${}^{4}$ bar at 1300 K). For these conditions, the solubility of nitrogen in liquid Ga attains relatively low values limited by $C_{N}<3\times 10^{-3}$ at. fr. For Fe these concentrations are much higher $C_{N}\leq 9\times 10^{-2}$ at. fr. The theoretical evaluations were clearly supported by available experimental results for both systems.

The solubility is temperature-dependent, as demonstrated by the relatively high enthalpy of dissolution, obtained from the concentration dependence which was $\Delta H_{therm}^{dis}=0.848\pm 0.001$ eV and $\Delta H_{therm}^{dis}=0.2889\pm 0.001$ eV for Ga and Fe, respectively. These values are different from the *ab initio* values which were $\Delta H_{dis}\left(\mathrm{Ga}-\frac{1}{2}N{}_{2}\right)=0.696$ eV and $\Delta H_{dis}\left(\mathrm{Fe}-\frac{1}{2}N{}_{2}\right)=0.172$ eV, respectively. The values following from the concentration dependence and the *ab initio* ones are considerably different, which confirms the important role of entropy contributions. It could be noted that much higher values of the enthalpy are observed for liquid Ga, which indicates the steeper temperature induced change of the N concentration for Ga. For Fe, the temperature dependence of the N concentration is much weaker what can be relevant for a proper design of the crystallization experiment where low supersaturation in this otherwise promising, growth solution has to be created.

## Data Availability

Data sharing is not applicable to this article.

## Sample Availability

Samples of the compound Fe with dissolved N are available from the authors.

## Appendix A. Singularity of Entropy Contribution to Solid-Vapor Transition at Zero K

Equilibrium between solid and vapor and entail chemical potential equality of both phases:

(A1) $$\mu {}_{v}\left(p,T\right)=\mu {}_{s}\left(p,T\right)$$

In general, the equality holds for any species exchanged between phases. Here for simplicity, we assume that single species is exchanged, thus for simplicity any reference is neglected. The chemical potential can be divided into enthalpy and entropy contributions, for both phases

(A2) $$\mu {}_{v,s}\left(p,T\right)=h_{v,s}\left(p,T\right)-Ts_{v,s}\left(p,T\right)$$

In ideal gas approximation, the pressure change of the chemical potential may be approximated as:

(A3) $$\mu {}_{v}\left(p,T\right)≅\mu {}_{s}\left(p_{0},T\right)+k_{B}Tln\left(\frac{p}{p_{0}}\right)$$

For solid phase, the specific volume is 4 orders of magnitude smaller, thus the solid phase contribution is neglected. Then the equilibrium pressure over the solid may be obtained as Van’t Hoff relation:

(A4) $$p≅p_{0}exp[-\frac{\Delta h_{vs}-T\Delta s_{vs}}{k_{B}T}]$$

where $\Delta h_{vs}=h_{v}-h_{s}>0$ and $\Delta s_{vs}=s_{v}-s_{s}>0$, are positively defined enthalpy and entropy of vaporization at normal conditions, respectively. Generally, these thermodynamic functions are temperature-dependent. In ideal gas approximation the specific heat $C_{p}$ is constant vs temperature, independent of the density (therefore a pressure could be used). The temperature dependence of the enthalpy of the vapor as:

(A5) $$h_{v}\left(T\right)=h_{v}\left(T_{0}\right)+C_{p}\left(T-T_{0}\right)$$

For the solid phase, the specific heat is given by Debye which, for low temperatures, may be approximated by cubic dependence $C_{s}\left(T\right)∼\alpha T^{3}$ which gives the following dependence of the enthalpy of solids at low temperatures

(A6) $$h_{s}\left(T\right)=h_{s}\left(T_{0}\right)+\alpha \left(T^{4}-T_{0}^{4}\right)/4$$

In summary, the enthalpies of both phases are regular at *T* = 0 K and application of zero K reference state for enthalpy is correct. Therefore, zero temperature is selected as the reference state, i.e., $T_{0}=0$ K.

Quite different conclusions are obtained for the entropy term. For the solid, the entropy contribution is regular at *T* = 0 K:

(A7) $$s_{s}\left(T\right)=s_{s}\left(T_{0}\right)+\int_{T_{0}}^{T}\frac{C(T)}{T}dT=s_{s}\left(T_{0}\right)+\alpha \left(T^{3}-T_{0}^{3}\right)/3$$

The vapor entropy term is singular, with logarithmic divergence at zero K:

(A8) $$s_{v}\left(T\right)=s_{v}\left(T_{0}\right)+\int_{T_{0}}^{T}\frac{C}{T}dT=s_{s}\left(T_{0}\right)+C_{p}ln\left(T/T_{0}\right)$$

Thus, the entropy at zero K cannot be used in determination of vapor–solid equilibria. Therefore, for entropy, normal pressure and temperature is selected: $p_{0}=1$ bar, $T_{0}=273.15$ K.

In general, the latter is a manifestation of well-known paradox, the entropy at zero K should be zero. For the ideal gas, the entropy is divergent, thus the vapor phase could not exist at zero K. It vanishes as the pressure of the vapor vanishes. In order to find the proper dependence of the vapor pressure at zero K the Van’t Hoff relation could be used (Equation (A4)). The precise relations is given by Equation (2), but for analysis of low temperature regime, the relation given by Equation (A4) is sufficient. Adopting above selection of zero temperature reference point for enthalpy, the enthalpy change is:

(A9) $$\Delta h_{vs}=\Delta H_{vap}\left(0\right)+C_{p}T-\alpha T^{4}/4$$

The entropy change could be approximated by

(A10) $$\Delta s_{vs}\left(T\right)=\Delta s_{vs}\left(T_{0}\right)+C_{p}\ln\left(T/T_{0}\right)-\alpha \left(T^{3}-T_{0}^{3}\right)/3$$

Hence, the vapor pressure, in equilibrium with the solid, at low temperatures may be approximated as:

(A11) $$p≅p_{0}(\frac{T}{T_{0}}{)}^{C_{p}/k_{B}}exp[-\frac{\Delta H_{vap}\left(0\right)}{k_{B}T}]exp\{\frac{1}{k_{B}}[\Delta s_{vs}\left(T_{0}\right)-C_{p}+\alpha (\frac{T_{0}^{3}}{3}-\frac{T^{3}}{12})]\}$$

At *T* = 0 K, the third term is constant while the first two vanish, confirming that ideal gas phase cannot exist at zero K. Therefore the agreement with the third principle of thermodynamics is confirmed.
